# Sequestration and Red Cell Deformability as Determinants of Hyperlactatemia in Falciparum Malaria

**DOI:** 10.1093/infdis/jiv502

**Published:** 2015-10-22

**Authors:** Haruhiko Ishioka, Aniruddha Ghose, Prakaykaew Charunwatthana, Richard Maude, Katherine Plewes, Hugh Kingston, Benjamas Intharabut, Charlie Woodrow, Kesinee Chotivanich, Abdullah Abu Sayeed, Mahtab Uddin Hasan, Nicholas P. Day, Abul Faiz, Nicholas J. White, Amir Hossain, Arjen M. Dondorp

**Affiliations:** 1 Mahidol Oxford Tropical Medicine Research Unit; 2 Department of Clinical Tropical Medicine, Faculty of Tropical Medicine, Mahidol University, Bangkok, Thailand; 3 Chittagong Medical College Hospital,; 4 Malaria Research Group and Dev Care Foundation, Dhaka, Bangladesh; 5 Nuffield Department of Clinical Medicine, Centre for Tropical Medicine and Global Health, University of Oxford, United Kingdom

**Keywords:** malaria, *Plasmodium falciparum*, hyperlactatemia, red blood cell deformability, parasite biomass

## Abstract

***Background.*** Hyperlactatemia is a strong predictor of mortality in severe falciparum malaria. Sequestered parasitized erythrocytes and reduced uninfected red blood cell deformability (RCD) compromise microcirculatory flow, leading to anaerobic glycolysis.

***Methods.*** In a cohort of patients with falciparum malaria hospitalized in Chittagong, Bangladesh, bulk RCD was measured using a laser diffraction technique, and parasite biomass was estimated from plasma concentrations of *Plasmodium falciparum* histidine-rich protein 2 (*Pf*HRP2). A multiple linear regression model was constructed to examine their associations with plasma lactate concentrations.

***Results.*** A total of 286 patients with falciparum malaria were studied, of whom 224 had severe malaria, and 70 died. Hyperlactatemia (lactate level, ≥4 mmol/L) was present in 111 cases. RCD at shear stresses of 1.7 Pa and 30 Pa was reduced significantly in patients who died, compared with survivors, individuals with uncomplicated malaria, or healthy individuals (*P* < .05, for all comparisons). Multiple linear regression analysis showed that the plasma *Pf*HRP2 level, parasitemia level, total bilirubin level, and RCD at a shear stress of 1.7 Pa were each independently correlated with plasma lactate concentrations (n = 278; *R*^2^ = 0.35).

***Conclusions.*** Sequestration of parasitized red blood cells and reduced RCD both contribute to decreased microcirculatory flow in severe disease.

Despite the recent gains in malaria control, falciparum malaria still causes an estimated 198 million cases globally and kills 0.58 million patients each year [1]. Once severe disease develops, the case-fatality rate is around 8% in children and 15% in adults despite optimal antimalarial treatment with artesunate [2]. A better understanding of the underlying pathophysiology is critical for developing adjunctive treatments to lower morality.

Lactic acidosis is a common complication of severe malaria, which has a strong predictive value for death in both pediatric and adult patients [3, 4]. Lactate accounts for approximately one fourth of the strong acids [5] present in severe malaria. The high lactate to pyruvate ratios in severe malaria indicate that anaerobic glycolysis most likely results from microcirculatory hypoperfusion [6]. Impairment of tissue microcirculatory flow is caused by the sequestration of cytoadherent red blood cells (RBCs) containing mature forms of the malaria parasite [7–9]. Rosetting, autoagglutination, and endothelial cell dysfunction may further compromise microcirculatory flow [10, 11]. Reduced uninfected RBC deformability (RCD) is thought to be an additional contributing factor [10, 12]. Other causes include circulatory shock and severe anemia, compromising oxygen delivery.

The relative contributions of these factors to lactic acidosis have not been well established. In this prospective study of adults hospitalized with falciparum malaria, we measured the plasma *P. falciparum* histidine-rich protein 2 (HRP2) concentration as a proxy measure for the total and sequestered parasite biomass [13], RCD, and other established contributors to lactic acidosis and assessed their relative associations with plasma lactate concentrations.

## PATIENTS AND METHODS

### Patients and Clinical Procedures

The study was performed at Chittagong Medical College Hospital in Bangladesh, which has a large catchment area extending to the Chittagong Hill Tracts. Falciparum malaria has low endemicity there, with a seasonal peak during the rainy season, from May until September. Consecutive patients admitted with falciparum malaria in the rainy seasons during 2005–2011 were included. The diagnosis of falciparum malaria was confirmed by microscopic examination of smears of peripheral blood specimens. Written informed consent was obtained from patients or attending relatives before enrollment. Severity of the disease was categorized according to modified World Health Organization criteria [14], including coma (Glasgow coma scale **<**11), pulmonary edema, repeated convulsions, severe anemia or jaundice (hematocrit **<**20% and bilirubin level of **>**2.5 mg/dL, combined with parasite counts of >100 000 parasites/µL), renal impairment (serum creatinine level of **>**3 mg/dL and/or anuria), hypoglycemia (blood glucose level of **<**40 mg/dL), shock (systolic blood pressure of **<**80 mm Hg with cool extremities), hyperparasitemia (peripheral asexual stage parasitemia level of **>**10%), hyperlactatemia (venous plasma lactate level of **>**4 mmol/L), or acidosis (venous plasma bicarbonate level of <15 mmol/L). A detailed medical history was recorded and a physical examination performed for all patients. Antimalarial treatment was with intravenous artesunate, and standard supportive care was given according to current guidelines [15, 16]. However, the availability of mechanical ventilation and renal replacement therapy was limited. Healthy individuals were recruited as a comparison group for assessment of RCD. Ethical approval for the study was obtained from the Bangladesh Medical Research Council and the Oxford Tropical Medicine Research Ethical Committee.

### Laboratory Procedures

On admission, venous blood samples were taken for thick and thin films, full blood count, routine biochemistry analysis, and plasma lactate levels. Plasma concentrations of lactate were measured in fluorooxalate anticoagulated samples, which were stored at −80°C until laboratory analysis by an Olympus analyzer during 2005–2010 and by a handheld automated analyzer (i-STAT, Abbott) during 2011. A predefined cutoff of 4 mmol/L was used to define hyperlactatemia [14]. Plasma concentrations of *Pf*HRP2 were measured in ethylenediaminetetraacetic acid–anticoagulated samples, which were stored at −80°C and transported on dry ice to the reference laboratory in Bangkok. *Pf*HRP2 concentration was measured using an enzyme-linked immunosorbent assay (Cellabs). RCD was measured shortly after venipuncture by ektacytometry, using a laser-assisted optical rotational cell analyzer (Mechatronics) [17]. RCD was assessed at a shear stress of 1.7 Pa and 30 Pa. Shear stresses of ≥1.7 Pa are encountered at the arterial side of the circulation and in the capillaries [18]. A high shear stress of 30 Pa is supraphysiological, although it might be encountered while traversing the sinusoids of the spleen, but changes at this stress reflect changes in volume to surface ratio. The ellipticity pattern obtained by laser diffraction through a RBC suspension under shear stress is directly proportional to the mean ellipticity of the RBCs. This ellipticity is described as the elongation index, defined by the ratio of the difference of the long axis and short axis of the ellipse and the sum of these 2 axes.

### Statistical Analysis

Differences in baseline demographic characteristics were compared between patients who died of and those who survived severe malaria, as well as between patients with malaria with and those without hyperlactatemia, using the χ^2^ test for categorical variables and the Student *t* test or Mann–Whitney *U* test for continuous variables. RCD values from patients who died from severe malaria were compared to patients who survived severe malaria, patients with uncomplicated malaria, and healthy participants, by analysis of variance, and comparisons between individual groups were corrected for multiple comparisons, using the Bonferroni test. Correlations between variables were assessed using the Spearman correlation coefficient. The variables associated with plasma lactate level were assessed using multiple linear regression analysis. Data were log transformed, if indicated. Variables in the final multivariate models were selected on the basis of their significance in the univariate analysis and their biological plausibility on the causal pathway from microcirculatory flow obstruction to hyperlactatemia. A 2-sided *P* value of <.05 was considered statistically significant. Analyses were performed using Stata, version 13.0 (StataCorp).

## RESULTS

### Baseline Characteristics

A total of 286 patients with falciparum malaria were included; 224 were classified as having severe malaria, of whom 70 died, and 62 patients had uncomplicated malaria, none of whom died. Fatal cases had significantly higher plasma lactate and lower plasma bicarbonate concentrations than survivors (Table 1). Systolic blood pressure on admission was higher in patients who died of severe malaria, compared with those who survived severe malaria. There was no significant difference in peripheral parasitemia or *Pf*HRP2 values between patients who died and those who survived. Six patients (2%) developed shock, defined by a systolic blood pressure of <80 mm Hg with cool extremities, and 2 (33%) died. Baseline characteristics of the patients are described in Table 1.

**Table 1.** Baseline Characteristics of Patients With Falciparum Malaria, Stratified by Disease Severity

Variable	Severe Malaria		Patients With Uncomplicated Malaria	*P* Value^a^
	Patients Who Died	Patients Who Survived		
Age, y	33.9 ± 13.7	34.5 ± 15.4	31.1 ± 15.0	.785
Male sex, patients, no. (%)	47 (67.1)	120 (77.9)	40 (64.5)	.086
Child aged <16 y, patients, no. (%)	3 (4.3)	7 (4.5)	6 (9.7)	.930
Temperature, °C	38.0 ± 1.3^b^	38.0 ± 1.2	37.7 ± 1.0^c^	.854
Respiratory rate, breaths/min	36 ± 9^b^	30 ± 10	27 ± 9^c^	<.001
Pulse rate, beats/min	116 ± 23	107 ± 19	94 ± 19	.003
Systolic blood pressure, mm Hg	117 ± 19^b^	111 ± 18	107 ± 16	.036
Diastolic blood pressure, mm Hg	68 ± 16^b^	66 ± 14	63 ± 11	.258
Glasgow coma scale	8 (6–10)	10 (8–14)	15 (15–15)^d^	<.001
White blood cell count, ×10^3^ cells/μL	10.5 (8.0–16.1)^e^	8.0 (6.3–10.4)^f^	6.2 (4.7–7.8)^g^	<.001
Hemoglobin level, g/dL	9.3 ± 3.0^f^	9.2 ± 2.5^h^	10.3 ± 2.9^d^	.865
Glucose level, mg/dL	116 (85–196)^b^	126 (95–160)^h^	110 (91–129)	.998
HCO_3_ level, mmol/L	15.3 ± 6.3^i^	18.9 ± 4.2^j^	22.9 ± 3.9	<.001
Lactate level, mmol/L	6.0 (4.0–9.7)	3.4 (2.3–4.9)	1.6 (1.3–2.2)	<.001
Creatinine level, mg/dL	1.5 (1.0–2.5)	1.4 (0.9–2.3)^k^	0.9 (0.8–1.1)^l^	.499
Total bilirubin level, mg/dL	4.7 (2.1–11.9)	2.6 (1.3–5.7)^k^	0.9 (0.6–2.3)^l^	.002
Parasitemia level, parasites/μL	93 070 (35 921–229 471)	85 848 (18 086–284 107)	27 004 (5275–74 606)	.436
*Pf*HRP2 level, ng/mL	2168 (908–5035)	2134 (764–4216)	428 (157–810)	.537

Data are mean ± SD or median (interquartile range) for 70 patients who died, 154 who survived, and 62 with uncomplicated malaria, unless otherwise indicated.

Abbreviations: HCO_3_, venous bicarbonate; *Pf*HRP2, *Plasmodium falciparum* histidine rich protein 2.

^a^ For comparison between patients with severe malaria who died vs those with severe malaria who survived, by the χ^2^ test, for categorical variables, and the Student *t* test or Mann–Whitney *U* test, for continuous variables.

^b^ Data are for 69 patients.

^c^ Data are for 60 patients.

^d^ Data are for 61 patients.

^e^ Data are for 65 patients.

^f^ Data are for 151 patients.

^g^ Data are for 58 patients.

^h^ Data are for 152 patients.

^i^ Data are for 68 patients.

^j^ Data are for 150 patients.

^k^ Data are for 153 patients.

^l^ Data are for 55 patients.

### RCD

RCD was reduced in proportion to disease severity (Table 2). RCD at both levels of shear stress (1.7 Pa and 30 Pa) were significantly different between patients who died of severe malaria, survivors of severe malaria, patients with uncomplicated malaria, and healthy individuals (*P* < .05, all).

**Table 2.** Admission Red Blood Cell Deformability (RCD) at Admission Among Patients With Falciparum Malaria and Healthy Controls

Variable	Study Group, Mean ± SD			
	Patients With Severe Malaria Who Died (n = 70)	Patients With Severe Malaria Who Survived (n = 154)	Patients With Uncomplicated Malaria (n = 62)	Healthy Controls (n = 39)
Age, y	33.9 ± 13.7	34.5 ± 15.4	31.1 ± 15.0	32.3 ± 10.0
Elongation index				
RCD at SS = 1.7 Pa	0.185 ± 0.033	0.197 ± 0.031	0.212 ± 0.027	0.233 ± 0.019
RCD at SS = 30 Pa	0.516 ± 0.035	0.534 ± 0.033	0.555 ± 0.028	0.581 ± 0.017

The Bonferroni correction was preapplied to all *P* values in 6 comparisons between 4 groups. RCD values at both levels of shear stress were significantly different between all study groups (*P* < .05, by analysis of variance).

Abbreviation: SS, shear stress.

### Factors Associated With Hyperlactatemia

Variables associated with hyperlactatemia were analyzed to construct multiple linear regression models including all patients with malaria (Table 3). There were 111 of 286 patients (38.8%) with hyperlactatemia (lactate level, ≥4 mmol/L), of whom 53 of 111 (47.7%) died, compared with 17 deaths among 175 patients (9.7%) without hyperlactatemia (*P* < .001). Systolic and diastolic blood pressure, hemoglobin concentration, and blood glucose level were not significantly different between patients with and those without hyperlactatemia (*P* = .597, *P* = .118, *P* = .134, and *P* = .280, respectively). Also, there was no significant difference in mean corpuscular volume between the 2 groups (*P* = .308), which is a contributor to variation in RCD. Among the variables significantly associated with hyperlactatemia, Glasgow coma scale and bicarbonate level were not included in the linear regression model, since they can be considered consequences of decreased microcirculatory flow, rather than causes. Total bilirubin and plasma creatinine levels were included in the model as crude proxy measures of hepatic and renal dysfunction contributing to hyperlactatemia. *Pf*HRP2, peripheral parasitemia, total bilirubin, and plasma creatinine levels and RCD at a shear stress 1.7 Pa were included in the final regression model. RCD at a shear stress of 30 Pa was excluded because of colinearity with RCD at a shear stress of 1.7 Pa (*r*_s_ = 0.52; *P* < .001). A shear stress of 1.7 Pa is considered to be encountered in the capillary bed, whereas 30 Pa is supraphysiological [18]. Previous studies have shown that RCD at a shear stress of 1.7 Pa has the strongest prognostic significance of a fatal outcome [12, 19]. Including interactions did not improve the model fit. There was a weak correlation between plasma *Pf*HRP2 level and RCD at shear stresses of 1.7 Pa (*r*_s_ = −0.23; *P* < .001) or 30 Pa (*r*_s_ = −0.27; *P* < .001).

**Table 3.** Clinical and Laboratory Characteristics of Patients With Falciparum Malaria, Stratified by Plasma Lactate Level

Variable	Lactate Level, ≥4 mmol/L	Lactate Level, <4 mmol/L	*P* Value^a^
Age, y	33.4 ± 13.8	33.8 ± 15.6	.840
Systolic blood pressure, mm Hg	112 ± 19^b^	111 ± 17	.597
Diastolic blood pressure, mm Hg	68 ± 15^b^	65 ± 14	.118
Glasgow coma scale	9 (6–11)	14 (9–15)^c^	<.001
Hemoglobin level, g/dL	9.2 ± 2.8^d^	9.7 ± 2.7^e^	.134
Corpuscular volume, fL	83.7 ± 7.8^f^	84.7 ± 8.1^g^	.308
HCO_3_ level, mmol/L	15.5 ± 5.0^d^	21.0 ± 4.4^h^	<.001
Glucose level, mg/dL	124 (88–185)^b^	114 (92–147)^h^	.280
Creatinine level, mg/dL	1.4 (1.0–2.5)	1.1 (0.8–1.8)^g^	.005
Total bilirubin level, mg/dL	4.1 (1.9–9.2)	1.8 (0.8–3.8)^g^	<.001
Parasitemia level, parasites/μL	137 155 (37 680–321 536)	42 201 (8290–159 638)	<.001
*Pf*HRP2 level, ng/mL	2633 (1090–5278)	816 (380–2611)	<.001
Elongation index			
RCD, SS = 1.7 Pa	0.187 ± 0.035	0.204 ± 0.028	<.001
RCD, SS = 30 Pa	0.524 ± 0.034	0.541 ± 0.034	<.001
Fatal outcome, patients, no. (%)	53 (47.7)	17 (9.7)	<.001

Data are mean ± SD or median (interquartile range) for 111 patients with a lactate level of ≥4 mmol/L and 175 with a lactate level of <4 mmol/L, unless otherwise indicated.

Abbreviations: HCO_3_, venous bicarbonate; *Pf*HRP2, *Plasmodium falciparum* histidine rich protein 2; RCD, red blood cell deformability; SS, shear stress.

^a^ By the χ^2^ test, for categorical variables, and the Student *t* test or Mann–Whitney *U* test, for continuous variables.

^b^ Data are for 110 patients.

^c^ Data are for 174 patients.

^d^ Data are for 107 patients.

^e^ Data are for 171 patients.

^f^ Data are for 102 patients.

^g^ Data are for 167 patients.

^h^ Data are for 173 patients.

**Figure 1.**
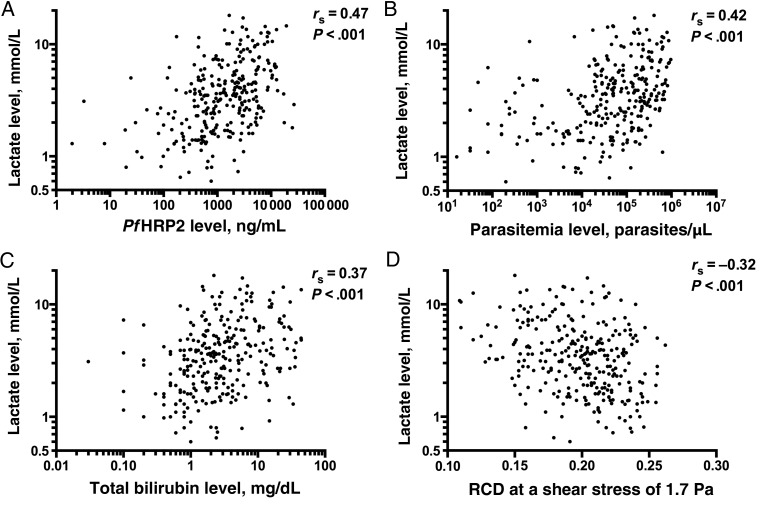
Univariate correlations of plasma lactate concentration with *Plasmodium falciparum* histidine-rich protein 2 (*Pf*HRP2) level (*A*), parasitemia level (*B*), total bilirubin level (*C*), and red blood cell deformability (RCD) at a shear stress of 1.7 Pa (*D*).

In the univariate analysis, levels of plasma *Pf*HRP2 (*r*_s_ = 0.47; *P* < .001), peripheral parasitemia (*r*_s_ = 0.42; *P* < .001), and total plasma bilirubin (*r*_s_ = 0.37; *P* < .001) were positively correlated with plasma lactate level (Figure 1*A*–*C*), while there was a negative correlation between RCD at a shear stress of 1.7 Pa and plasma lactate level (*r*_s_ = −0.32; *P* < .001; Figure 1*D*). RCD at a shear stress of 30 Pa was not included in the regression model but was also inversely correlated with plasma lactate level (*r*_s_ = −0.35; *P* < .001). Compared with other variables, plasma creatinine level had a weak correlation with plasma lactate level (*r*_s_ = 0.22; *P* < .001). The multiple linear regression model was constructed including 278 patients. The variance inflation factors of all variables were <2. Residual analyses indicated no substantial deviation from model assumptions. The model showed that plasma *Pf*HRP2, peripheral parasitemia, and total bilirubin levels and RCD at a shear stress of 1.7 Pa were all independently associated with plasma lactate concentrations (Table 4). The overall predictive value of the model regarding plasma lactate showed an *R*^2^ of 0.35. Including MCV in the regression model did not change the results (n = 264; *R*^2^ = 0.35).

**Table 4.** Multiple Linear Regression Analysis of Variables Associated With Plasma Lactate Concentration in Patients With Falciparum Malaria

Variable	β^a^ (95% CI)	*P* Value
*Pf*HRP2 level^b^	0.293 (.180–.406)	<.001
Parasitemia level^b^	0.251 (.148–.355)	<.001
Total bilirubin level^b^	0.169 (.060–.278)	.003
Plasma creatinine level^b^	−0.021 (−.134 to .091)	.707
RCD at SS of 1.7 Pa, EI	−0.177 (−.279 to −.075)	.001
R^2^	0.346	
Adjusted R^2^	0.334	

The final regression model was based on 278 patients.

The other variables included in the model were: systolic and diastolic blood pressure, hemoglobin concentration, blood glucose, and MCV.

Abbreviations: CI, confidence intervals; EI, elongation index; *Pf*HRP2, *Plasmodium falciparum* histidine-rich protein 2; RCD, red blood cell deformability; SS, shear stress.

^a^ Standardized partial regression coefficient.

^b^ Analyzed on a log scale.

## DISCUSSION

The major determinant of reduced microcirculatory flow in severe falciparum malaria is sequestration caused by cytoadherance of parasitized RBCs to the vascular endothelium, which reduces the luminal diameter of capillaries and postcapillary venules. It has been proposed that, in addition, microcirculatory flow is variably compounded by the adherence of parasitized RBCs to uninfected RBCs (rosette formation), agglutination of parasitized RBCs (autoagglutination), endothelial dysfunction leading to disordered vascular tone, and reduced RCD. Rosette formation and autoagglutination are in vitro phenomena, demonstrated by culture of parasite field strains, which was not included in this study. Endothelial dysfunction is difficult to quantify and was also not part of our analysis. Other studies have shown a relationship between the extent of nitric oxide–mediated hyperemia, measured as pulse wave amplitude, after bloodlessness (EndoPAT, Itamar Medical) and plasma lactate levels in severe malaria. In this study, both the *Pf*HRP2 level, reflecting total parasite biomass, and the RCD at shear stresses of 1.7 Pa and 30 Pa significantly correlated with plasma lactate concentration, both in the univariate and multivariate analysis. Blood pressure, plasma glucose level, and hemoglobin concentration did not correlate with plasma lactate level. Plasma creatinine level, as a measure of renal function, weakly correlated with plasma lactate level, whereas plasma total bilirubin level, as a measure of liver function, showed a stronger correlation with plasma lactate level and contributed significantly to the multivariate model. These results support the concept that both parasitized RBC sequestration and a reduction in RCD contribute to reduced microcirculatory flow in falciparum malaria. Reduced RCD is a pathophysiological feature also observed in hematological diseases such as sickle cell disease, hereditary spherocytosis, and thalassemia [20], while the increase in lactate level is not a typical manifestation of these diseases. This suggests that reduced RCD on its own is not sufficient to cause a significant reduction in tissue perfusion but that a reduction in RCD does contribute when capillary diameter is reduced by cytoadherent rigid parasitized RBCs.

The main determinants of RCD are the geometric properties of RBCs, cytoplasmic viscosity, and rigidity of the RBC membrane, all of which are impaired in parasitized RBCs in falciparum malaria infection. The surface area to volume ratio is reduced and sphericity is increased in parasitized RBCs [21]. As the intra-erythrocytic parasite matures to the trophozoite and schizont stages, both the internal viscosity and membrane rigidity of the parasitized RBCs increase [22, 23]. However, in severe falciparum malaria the RCD of uninfected RBCs is also reduced [17, 24]. With ektacytometry, fractions with different RCDs contribute according to their size to the overall RBC bulk. Since the majority of RBCs in falciparum malaria are uninfected, the reduction in RCD is mainly caused by rigidity of uninfected RBCs. The presence of a large population of the parasitized RBCs could contribute to a reduction in RCD. In our study, hyperparasitemia (peripheral asexual stage parasitemia level, **>**10%) was present in 44 of 286 (15.4%). In these patients, the contribution of the parasitized RBCs is still relatively small (proportional to the parasitemia percentage). Increased membrane rigidity is one of the potential contributors to the rigidity of the uninfected RBCs [12]. This could be caused by oxidative damage mediated by cell-free hemoglobin or other parasite products, but this needs further study. In the current study, we found a correlation between plasma *Pf*HRP2 level as a measure of the total parasite biomass and RCD, although this correlation was weak.

The replacement of parasitized RBCs and rigid uninfected RBCs with RBCs of normal deformability could have beneficial effect on the restoration of RCD in severe malaria. A previous study indeed showed that blood transfusion improved RCD of African children with severe falciparum malaria [12]. Exchange transfusion can be expected to have a larger effect on the restoration of RCD. However, a recent large case control study showed no survival benefit from exchange transfusion in returning travelers with severe falciparum malaria [25]. The efficacy of exchange transfusion could be decreased due to the reduction of deformability of stored allogeneic RBCs [26].

In addition to the RCD and estimated total parasite burden, peripheral parasitemia and total bilirubin levels correlated with plasma lactate concentrations in the multivariate model. Since the total parasite burden estimate from the plasma *Pf*HRP2 level denotes the burden present in the previous parasite cycle, peripheral blood parasitemia level could be a proxy measure for the additional total burden at the moment of blood collection. The correlation with plasma bilirubin level could indicate liver dysfunction affecting lactate clearance. The multivariate linear regression model including these 4 variables explained 35% of the variation in plasma lactate level, suggesting that additional factors contribute to the pathogenesis of hyperlactatemia. D-lactate derived from parasite represents a portion of the lactate level during severe malaria, but the contribution is estimated as 6%–7% of the total lactate level [27].

In conclusion, high plasma concentrations of *Pf*HRP2 and reduced RCD correlated with hyperlactatemia in patients with falciparum malaria. This suggests that both sequestration of parasitized RBCs and reduced RCD contribute to decrease microcirculatory flow in severe cases of malaria.
